# Effects of Lactone- and Ketone-Brassinosteroids of the 28-Homobrassinolide Series on Barley Plants under Water Deficit

**DOI:** 10.3390/plants13101345

**Published:** 2024-05-13

**Authors:** Liliya V. Kolomeichuk, Ol’ga K. Murgan, Elena D. Danilova, Mariya V. Serafimovich, Vladimir A. Khripach, Raisa P. Litvinovskaya, Alina L. Sauchuk, Daria V. Denisiuk, Vladimir N. Zhabinskii, Vladimir V. Kuznetsov, Marina V. Efimova

**Affiliations:** 1 Department of Plant Physiology, Biotechnology and Bioinformatics, Biological Institute, National Research Tomsk State University, Lenin Avenue 36, Tomsk 634050, Russia; kolomeychuklv@mail.ru (L.V.K.); reborn_rinni@mail.ru (O.K.M.); danilova_lenochk@mail.ru (E.D.D.); masha_serafimovich@mail.ru (M.V.S.); 2 Institute of Bioorganic Chemistry, National Academy of Sciences of Belarus, Kuprevich Street 5/2, 220084 Minsk, Belarus; khripach@iboch.by (V.A.K.); alina_kyrtikova@mail.ru (A.L.S.); vz@iboch.by (V.N.Z.); 3 K.A. Timiryazev Institute of Plant Physiology, Russian Academy of Sciences, Botanicheskaya Street 35, Moscow 127276, Russia; vlkuzn@mail.ru

**Keywords:** antioxidant enzymes, endogenous content of brassinosteroids, gene expression, *Hordeum vulgare* L., photosystem II, photosynthetic pigments, proline, water status

## Abstract

The aim of this work was to study the ability of 28-homobrassinolide (HBL) and 28-homocastasterone (HCS) to increase the resistance of barley (*Hordeum vulgare* L.) plants to drought and to alter their endogenous brassinosteroid status. Germinated barley seeds were treated with 0.1 nM HBL or HCS solutions for two hours. A water deficit was created by stopping the watering of 7-day-old plants for the next two weeks. Plants responded to drought through growth inhibition, impaired water status, increased lipid peroxidation, differential effects on antioxidant enzymes, intense proline accumulation, altered expression of genes involved in metabolism, and decreased endogenous contents of hormones (28-homobrassinolide, B-ketones, and B-lactones). Pretreatment of plants with HBL reduced the inhibitory effect of drought on fresh and dry biomass accumulation and relative water content, whereas HCS partially reversed the negative effect of drought on fresh biomass accumulation, reduced the intensity of lipid peroxidation, and increased the osmotic potential. Compared with drought stress alone, pretreatment of plants with HCS or HBL followed by drought increased superoxide dismutase activity sevenfold or threefold and catalase activity (by 36%). The short-term action of HBL and HCS in subsequent drought conditions partially restored the endogenous B-ketone and B-lactone contents. Thus, the steroidal phytohormones HBL and HCS increased barley plant resistance to subsequent drought, showing some specificity of action.

## 1. Introduction

Drought, as one of the main negative factors of global climate change, causes enormous economic damage to agricultural production and thus makes it difficult to fight hunger [1,2]. For example, according to NOAA’s National Centers for Environmental Information (NCEI) in the USA, from 1980 to 2020, there were 26 severe droughts in the U.S., with damage totaling at least USD 249 billion [3].

Plants respond to water deficits through a range of morphological, physiological, and molecular reactions [1]. It has been shown that the contents of the main photosynthetic pigments, functional activity of photosystem II, and water status are reduced under drought conditions [2]. Simultaneously, the generation of reactive oxygen species (ROS) increases, the intensity of lipid peroxidation increases [4], and the activities of antioxidant enzymes and the levels of low-molecular-weight organic compounds with antioxidant properties increase [5,6].

The development of effective technologies is necessary to protect plants from the negative effects of drought. A very promising way to protect plants from abiotic stresses, including drought, is the use of growth regulators. To date, methods for increasing plant resistance to water deficit have been developed using glycine betaine [7], folic acid [6], α-lipoic acid, cysteine [8], and others. It is widely known that the increase in plant resistance is largely determined by factors of hormonal nature [9,10]. In this regard, brassinosteroids (BRs) are of particular interest because they are involved in the regulation of plant growth and ontogenesis and, by acting at extremely low concentrations, exhibit a pronounced stress-protective effect [10,11,12].

To increase plant resistance to the action of damaging factors, hormonal treatment is carried out during or before the action of the stressor [13,14,15]. Recently, short-term pretreatment of seeds or plants with phytohormones that initiate a priming state has attracted much interest [16,17]. Priming is defined as the process by which an organism acquires the ability to increase stress tolerance in response to some damaging factor in the future. Factors of different natures, including BRs, can serve as inducers of the plant transition to the priming state [17,18,19,20].

Earlier, we showed that short-term root treatment of rape and potato plants with BRs increased their stress tolerance to subsequent chloride salinization. Treatment of plants with lactone-containing BRs (24-epibrassinolide (EBL) and 28-homobrassinolide (HBL)) induced the transition of potato plants to the priming state, which was manifested in the ability of plants to respond to subsequent salt stress by more effective accumulation of proline and carotenoids with pronounced antioxidant and stress-protective properties [11,18]. Pretreatment of rapeseed plants with lactone-containing EBL and ketone-containing 24-epicastasterone under subsequent salt stress increased the activity of the antioxidant enzymes superoxide dismutase (SOD) and guaiacol peroxidase. This pretreatment reduced the influx of sodium and chlorine ions into the root system under saline conditions, the consequence of which was a decrease in the osmotic potential of cells and maintenance of water status [21].

Despite the publication of a large number of articles on the protective role of BRs under stress conditions, including drought, the data of different authors on the response of plants to the action of BRs are very dynamic, if not contradictory, which is determined by the specifics of the object, the nature of the BRs used, the intensity and duration of drought, and the conditions of hormonal treatment [14]. At the same time, in the vast majority of cases, EBL was used almost exclusively for plant treatment under drought conditions [13,14,20], while the stress-protective effects of other groups of BRs still remain unclear [12]. This is explained by the fact that lactone-containing compounds of a number of BRs (EBL, HBL) are easily available to the scientific community and agricultural producers (in the form of agropreparations), and the mechanism of their protective effect is being actively studied, whereas ketone-containing BRs are currently much less available, and the mechanism of their action is poorly understood.

It is also important to note that in response to drought and/or exogenous BRs with subsequent water deficit, plants respond by changing the endogenous content of BRs, which may be relevant to the realization of their protective effect [22,23]. As a rule, the total content of BRs or the content of only brassinolide and/or castasterone has been studied, although there are a few exceptions [14,24].

However, the response of plants, including monocotyledons such as barley, to the action of steroidal phytohormones of different chemical structures at the physiological function and endogenous BR content levels has not been studied in practice. A demonstration of the protective effect of ketone-containing BRs under drought conditions would allow us to expand the range of practically relevant representatives of steroid phytohormones.

The aim of this work was to compare the ability of steroidal phytohormones with different chemical structures, namely lactone-containing (HBL) and ketone-containing (28-homocastasterone, HCS) BRs, to increase drought tolerance in barley plants and to alter the status of endogenous brassinosteroids.

## 2. Results

### 2.1. Growth Parameters, Water Contents, and Leaf Cell Sap Osmotic Potential

**Figure 1:**
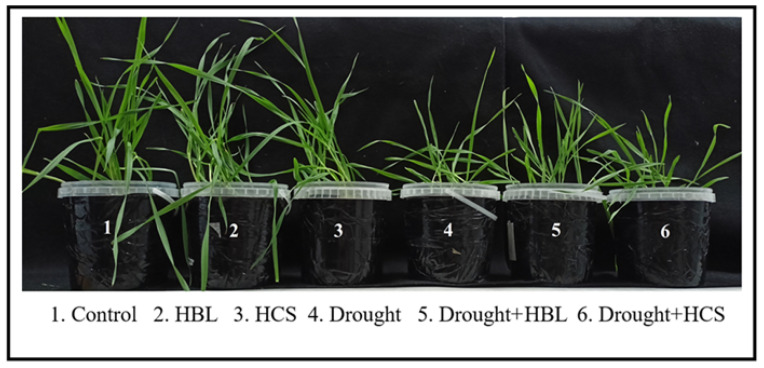
Effect of short-term (2 h) plant pretreatment with HBL and HCS on biomass accumulation by barley plants under normal conditions and under water deficit conditions (14 days).

The effects of plant pretreatment with HBL and HCS on the physiological parameters of barley during subsequent drought stress were evaluated. Under optimal growing conditions, the pretreatment of plants with HCS slightly (by 8 and 18%) increased the fresh and dry biomass of plants, respectively, relative to those of the control (Table 1, Figure 1). Under drought conditions, decreases of 43% and 24% in plant fresh and dry weight, respectively, and 20% in relative water content were observed compared with those in the control. Pretreatment of plants with HBL significantly reduced the inhibitory effect of drought on the accumulation of fresh and dry weight and relative water content of plants, whereas the pretreatment of plants with HCS partially relieved the negative effects of water deficit on plant fresh weight under subsequent drought conditions.

**Figure 2:**
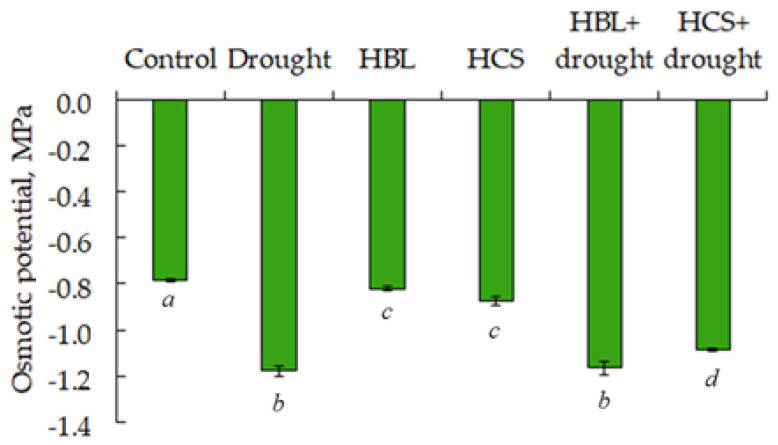
Effects of HBL, HCS, and drought on the osmotic potential of the cell contents of barley plant leaves. Values are given as the mean ± SD for each treatment. Values not sharing a common or same alphabet letter (*a*–*d*), and they differ significantly at *p* < 0.05 (Duncan’s multiple range test).

Fundamentally important for plant survival under drought conditions is the preservation of the organism’s water status [25]. As follows from the data presented in Figure 2, the osmotic potential of cell exudate of barley leaves under drought and in the case of pretreatment of plants with HBL with subsequent drought decreased by 1.5 times relative to the control. In the case of the pretreatment of HCS plants with subsequent drought, a slight increase in osmotic potential was observed compared with plants exposed to only one water deficit (Figure 2). At the same time, plants treated with BRs under normal conditions slightly decreased the level of osmotic potential of cell contents compared with the control (Figure 2).

### 2.2. Contents of Basic Photosynthetic Pigments and Primary Photosynthetic Processes

In addition to the effect of water deficit on biomass accumulation, the negative effects of drought were also reflected in the content of photosynthetic pigments (chlorophylls *a* and *b*, carotenoids) in barley leaves, which decreased by 28–35% relative to that in the control (Table 2). It cannot be excluded that the decrease in the level of pigments under drought conditions can be associated not only with their degradation under stress but also with the inhibition of their synthesis, which may be an adaptive response of plants to minimize the formation of ROS in photosynthetic membranes under moisture deficiency, as well as to optimize the use of light energy for photosynthetic processes [26]. Pretreatment of plants with hormones under normal conditions had almost no effect on the levels of major photosynthetic pigments, whereas the pretreatment of plants with HBL enhanced the water-deficit-induced decrease in photosynthetic pigments. Moreover, the exposure of plants to HCSs slightly ameliorated the inhibitory effect of drought on the chlorophyll *a* and *b* contents but not on the carotenoid content.

In addition to studying the effect of BRs on the content of the main photosynthetic pigments under subsequent drought, the functional activity of the photosynthetic apparatus was investigated by chlorophyll *a* fluorescence using a PAM fluorimeter. The parameters Fv/Fm (maximum quantum efficiency of photosystem II, PS II); ETR (electron transport rate); Y(II) (photochemical utilization of excitation energy in photosystem II under active light); qP (photochemical fluorescence quenching coefficient); and NPQ (nonphotochemical fluorescence quenching) were evaluated. As shown in Table 3, neither water deficit nor prehormone treatment of plants followed by drought significantly affected the investigated chlorophyll fluorescence parameters.

### 2.3. The Content of Lipid Peroxidation Products

**Figure 3:**
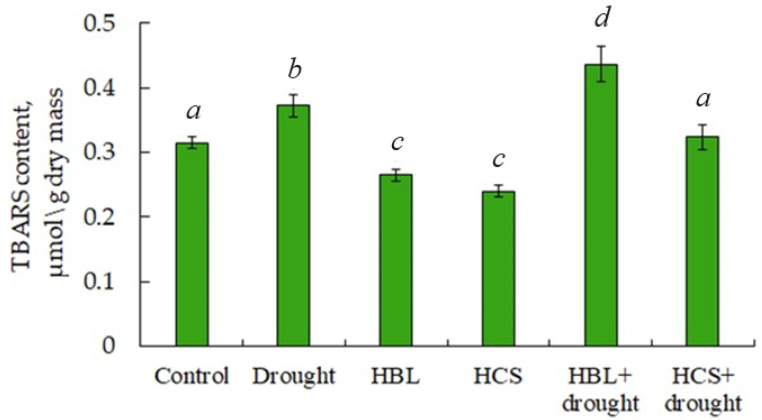
Effect of HBL, HCS, and drought on the content of TBARS-active products in leaves of barley plants. Values are given as the mean ± SD for each treatment. Values not sharing a common or same alphabet letter (*a*–*d*), and they differ significantly at *p* < 0.05 (Duncan’s multiple range test).

The magnitude of lipid peroxidation in barley plants was estimated by the content of products active in reaction with thiobarbituric acid (TBARS) during heating. Under optimal growth conditions, pretreatment of plants with HBL and HCS reduced the TBARS content by 16% and 24%, respectively, compared with that of the control (Figure 3). Under drought conditions, the TBARS content in leaves exceeded the control level by 19%. Pretreatment of plants with hormones followed by stress resulted in a 20% increase in TBARS levels in the case of HBL and, in contrast, in the removal of the drought-induced increase in TBARS content in the case of HCS (Figure 3).

### 2.4. Activity of Antioxidant Enzymes

**Figure 4:**
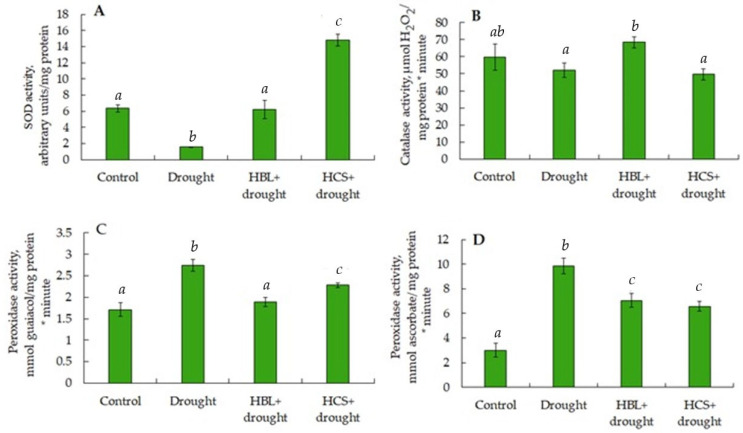
Effect of HBL, HCS, and drought on superoxide dismutase (**A**), catalase (**B**), guaiacol-dependent peroxidase (**C**), and ascorbate peroxidase (**D**) activities in leaves of barley plants. Values are given as the mean ± SD for each treatment. Values not sharing a common or same alphabet letter (*a*–*c*), and they differ significantly at *p* < 0.05 (Duncan’s multiple range test).

To reduce ROS levels and prevent the development of oxidative stress in plants, antioxidant defense systems are activated, the action of which is aimed at quenching reactive oxygen species. SOD, CAT, guaiacol-dependent peroxidase, and ascorbate-dependent peroxidase are important enzymes involved in antioxidant defense. As shown in Figure 4, in response to drought, there was a 3-fold decrease in SOD activity, a 3.2-fold increase in ascorbate-dependent peroxidase activity, and a 60% increase in guaiacol-dependent peroxidase activity in barley leaves relative to those in the control. Pretreatment of plants with HCSs followed by drought increased SOD activity sevenfold, did not affect catalase activity, and slightly decreased peroxidase activity relative to those under water deficit conditions. Pretreatment of plants with HBL increased SOD activity threefold, increased catalase activity by 36%, and decreased peroxidase activity relative to the effect of one drought (Figure 4).

### 2.5. Accumulation of Proline and Expression of Genes Related to Proline Metabolism

**Figure 5:**
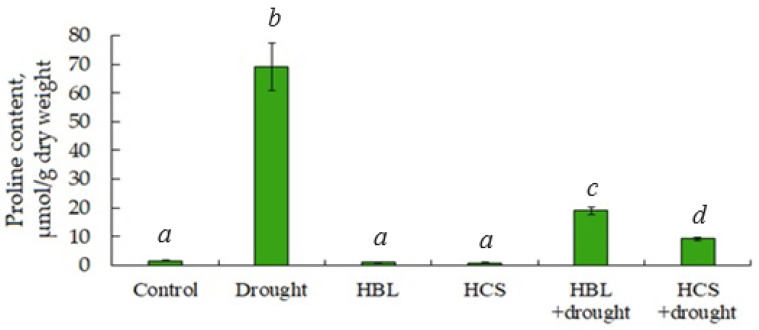
Effect of HBL, HCS, and drought on proline content in leaves of barley plants. Values are given as the mean ± SD for each treatment. Values not sharing a common or same alphabet letter (*a*–*d*), and they differ significantly at *p* < 0.05 (Duncan’s multiple range test).

One of the mechanisms of plant defense against abiotic stress is the accumulation of low-molecular-weight organic compounds, such as the imino acid proline, which acts as a chemical chaperone, osmoprotectant, metal chelator, and antioxidant [27,28]. Our data showed that pretreatment of plants with BRs did not affect the level of proline in leaves when barley was grown under normal moisture conditions (Figure 5). Under conditions of water deficit, the increase in proline content in barley leaves was 57.9 times greater than that in the control. Pretreatment of plants with BRs followed by drought resulted in significant inhibition of proline accumulation induced by water deficit. However, even in this case, its content was 16.4-fold and 9.5-fold greater in plants pretreated with HBL or HCS followed by drought, respectively, than in the control plants (Figure 5).

The key enzymes of the glutamate pathway of proline synthesis are pyrroline-5-carboxylate synthase (P5CS1) and pyrroline-5-carboxylate reductase (P5CR). Proline degradation is carried out by proline dehydrogenase (*PDH*). Evaluation of the transcript levels of proline metabolism genes revealed that in response to drought, plants respond by stimulating the expression of proline biosynthesis genes (*P5CS1* and *P5CR*) and inhibiting the activity of its degradation gene (*PDH*), resulting in intensive stress-dependent proline accumulation. HBL under normal growth conditions activated the accumulation of *P5CS1* gene transcripts, whereas HCS increased the levels of *P5CS1* and *P5CR* gene transcripts relative to those in control plants. Under the effect of subsequent drought, HCS-treated plants showed less accumulation of *P5CS1* transcripts but greater *P5CR*. Pretreatment of plants with HBL followed by drought reduced the level of *P5CR* gene transcripts but had no effect on *P5CS1* gene expression (Table 4, Table S1).

Next, we evaluated the expression level of the *P5CDH* gene encoding pyrroline-5-carboxylate dehydrogenase. This enzyme is involved in proline degradation following proline dehydrogenase. Contrary to expectations about unidirectional changes in the intensity of expression of two proline degradation genes (*P5CDH* and *PDH*), an increase in the level of *P5CDH* gene transcripts was observed under drought (5-fold), as well as under BR pretreatment of plants under normal growing conditions (two–threefold). However, when plants were pretreated with HBL and HCS followed by a 2-week drought, the level of *P5CDH* gene transcripts relative to water deficit was downregulated, possibly contributing to proline accumulation (Table 4, Table S1).

### 2.6. Brassinosteroid Content under Drought Conditions and BR Pretreatment

**Figure 6:**
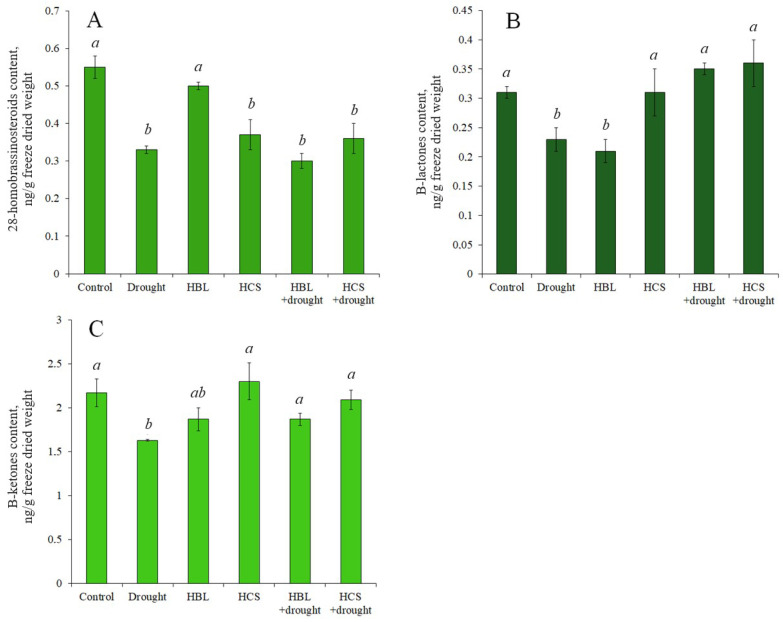
Content of 28-homobrassinosteroids (**A**), B-lactones (**B**), and B-ketones (**C**) in barley plants of “Biom” variety subjected to drought and/or exogenous treatment with 28-homobrassinosteroids. Values are given as the mean ± SD for each treatment. Values not sharing a common or same alphabet letter (*a*,*b*), and they differ significantly at *p* < 0.05 (Duncan’s multiple range test).

The contents of endogenous BRs of 28-homobrassinosteroids (HBL, HCS, 6-deoxo- HCS), B-lactones (brassinolide, 24-epibrassinolide, HBL, 28-norbrassinolide), and B-ketones (castasterone, HCS, 24-epicastasterone, 28-norcasterone) were determined (Figure 6). Under drought conditions, a decrease in the level of all determined BRs relative to the control was observed: for 28-homobrassinosteroids, it was the most significant and amounted to 40%, and for B-lactones and B-ketones, it was 25–26%. Treatment with HBL at a concentration of 0.1 nM resulted in a decrease in the level of 28-homobrassinosteroids (10%) relative to the control, B-lactones and B-ketones (33 and 14%, respectively). HCS treatment resulted in a more dramatic decrease in 28-homobrassinosteroids (33%), while the content of B-lactones was practically unchanged, and B-ketones slightly increased (6%). Pretreatment of plants with HBL and HCS followed by drought did not significantly change the level of BRs in the 28-homobrasinosteroid series, increasing the content of B-ketones compared with that under drought conditions by 15 and 28%, respectively, and the content of B-lactones by 56%, respectively, in the case of pretreatment of plants with HCS, fully compensating for the negative effect of drought.

## 3. Discussion

Brassinosteroids are involved in the regulation of differential genome expression, cellular metabolism, and integral physiological processes. Of particular interest is their ability to increase plant resistance to damaging abiotic factors of various physical natures, such as drought. The protective effect of exogenous BRs under conditions of water deficit has been convincingly demonstrated in many plant species [10,29]. However, whether the stress-protective effect of steroidal phytohormones depends on their chemical structure remains poorly investigated. The experimental data obtained allowed us to discuss the specificity of the action of BRs with different chemical structures (HBL and HCS) on barley plants under conditions of soil drought and whether BRs affect the endogenous content of 28-homoBS, B-ketones, and B-lactones.

### 3.1. Plant Growth and Photosynthesis under Drought and 28-Homobrassinosteroid Pretreatments

One of the main negative effects of drought is the inhibition of plant growth, which is based on the development of water deficit and a decrease in water potential. The application of exogenous BRs is known to contribute to the maintenance of water potential in plants under stress conditions [30]. This effect depends on the method of plant hormonal treatment. For example, treatment of flax-folk plants with HBL during drought did not affect the level of plant water potential, but pretreatment of the plants with hormones before sowing led to an increase in the water-holding capacity of the leaves of the flax seedlings [31].

Interestingly, the water status of plants depends not only on the method of hormone treatment but also on the endogenous level of BRs. Chen et al. [32] showed that the brassinosteroid-deficient cotton *pag1* mutant was characterized by low productivity and drought tolerance, which was due to its extremely poor tissue water-holding capacity, apparently due to reduced ABA levels and inability to regulate the transpiration rate. Treatment of sorghum plants with brassinosteroids stimulated the growth of the root system and thus increased its water-holding capacity and reduced water loss through transpiration [33].

In our study, drought resulted in a 43% and 24% decrease in the fresh and dry weight of the aboveground parts of barley plants, respectively, and a 20% decrease in the relative water content compared with that of the control. A significant decrease in the osmotic potential of the cellular exudate in the leaves was observed (Table 1). Pretreatment of plants with BRs resulted in partial removal of the negative effects of drought on the biomass accumulation and relative water content of barley plants. Moreover, these effects depend on the chemical structure of the BRs used. Thus, HBL reduced the inhibitory effect of drought on the accumulation of wet and dry biomass and relative water content without affecting the water potential of leaf cells. Moreover, pretreatment of plants with HCS partially relieved the negative effect of drought on fresh biomass accumulation and increased the osmotic potential by 40% but had no effect on plant dry mass or relative water content (Table 1, Figure 2). This effect could be due to hormonal regulation of ion accumulation in different parts of plants.

It could not be excluded that the stress-protective effect of BRs on plant biomass accumulation could be due to their effect on photosynthetic rate under drought conditions. We studied the effects of drought and hormone pretreatment of barley plants followed by drought stress on the photochemical activity of PSII and the content of main photosynthetic pigments.

The data obtained showed that neither drought nor pretreatment of plants with BRs followed by drought had a significant effect on the parameters of PSII photochemical processes (Table 3), although Fv/Fm values have been successfully used in many cases to assess “stress” in plants, reflecting the high sensitivity of PSII to environmental stimuli [34]. However, the interpretation of this index is not always unambiguous, because inactivation of PSII reaction centers is usually associated with heat loss and a decrease in Fv/Fm. Moreover, the optical properties of a leaf can change under certain stress conditions such as drought, which means that Fm and Fo values may be the result of changes in leaf absorptive capacity [34]. According to Baker and Rosenqvist (2004), stomatal closures did not correlate to Fv/Fm under mild drought conditions, and Fv/Fm itself is not a good indicator for detecting plant drought response at mild soil water deficit [35]. It was also found that Fv/Fm values do not change with elevated carbon dioxide under drought conditions [36]. It should be noted that in recent years, the hypothesis of relative resistance to water stress of both PSI and PSII has been experimentally confirmed. Thus, under the influence of soil drought, the ratio between the accumulation of photochemically inactive reaction centers of PSI and PSII practically does not change, which indicates the stability of the electron-transport chain of photosynthesis [37].

We also cannot exclude that the absence of Fv/Fm changes in barley under water deficit may reflect the specificity of this plant. This is evidenced by our preliminary experiments on the barley cultivar used in this work, the results of which indicate the stability of PSII photochemical processes under salt stress (unpublished data). This is also supported by the fact that in different barley cultivars, Fv/Fm values are independent of the intensity of light stress [38]. According to the literature, barley and oilseed rape seedlings that are exposed to exogenous BRs under optimal growth conditions showed no change in the efficiency of energy flow in PSII during prompt fluorescence measurements [39]. The above analysis suggests that the functional stability of PSII of young barley plants under drought is determined by the specificity of this species and/or the intensity of water stress.

It is known that plant productivity can be determined by the photosynthetic activity of the assimilation apparatus, which in turn depends on the content of the main photosynthetic pigments [40]. In the present work, a 28–35% decrease in pigment content relative to that of the control was observed against the background of drought, which was previously demonstrated in other plants [41]. Pretreatment of plants with HCS did not affect the content of chlorophylls and carotenoids under subsequent drought, whereas pretreatment with HBL resulted in a slight (10–15%) decrease in the content of photosynthetic pigments (Table 2). These data allow us to discuss the question of how BR, without increasing the content of photosynthetic pigments, reduces the negative effect of drought on plant biomass accumulation.

In answering this question, first of all, it should be noted that there is no direct relationship between pigment content and photosynthetic rate in plants, as evidenced by a number of facts. Tang et al. (2023) obtained mutants of *Setaria italic* that had significantly decreased Chl *b* content but increased photosynthetic rate; another group of mutants showed a positive correlation between increased stomatal conductance and increased photosynthetic rate and a weak correlation between decreased Chl *b* content and decreased photosynthetic rate [42]. BR treatment of rhododendron plants under drought was accompanied by an increase in photosynthetic rate, stomatal conductance, excitation energy capture efficiency of the reaction center, photochemical efficiency of PSII, photochemical quenching, and electron transport rate without significant effects on photosynthetic pigment content [43]. The BS mimic αDHECD was shown to increase photosynthetic rate under normal temperature conditions. Under heat stress conditions, αDHECD increased photosynthetic rate, stomatal conductance, and transpiration rate, while decreasing intercellular CO_2_ concentration and water use efficiency. Finally, the light absorbed by the mutant rice with reduced chlorophyll content was more efficiently partitioned to photosynthesis and could be used to improve photosynthetic efficiency [44].

The lack of a direct relationship between pigment content and photosynthetic rate suggests that HBL and HCS affect photosynthetic rate not through the stimulation of chlorophyll synthesis but through effects on other processes, such as stomatal conductance, which accelerates carbon dioxide uptake and increases photosynthetic rate. This assumption is entirely plausible, as there are many published articles showing, for example, that BS-induced improvements in CO_2_ assimilation under drought conditions are mainly due to stomatal events [45]. This is also supported by data from Junior et al. (2020), who found that BR treatment increased the photosynthetic rate and stomatal conductance to be involved in drought stress response in *Eucalyptus urophylla* [46].

### 3.2. Plant Oxidative Status under Drought and 28-Homobrassinosteroid Pretreatments

Plants respond to unfavorable environmental conditions, including drought, by accumulating ROS and developing oxidative stress [47]. The formation of ROS is caused by the “overload” of the electron-transport chain of chloroplasts under conditions of water deficit, which is associated with a decrease in the intensity of CO_2_ fixation and activation of the respiration process [48]. ROS cause the degradation of proteins and nucleic acids, oxidation of membrane lipids, breakdown of photosynthetic pigments, and inactivation of enzyme systems. BRs can reduce the negative effects of drought by activating the cellular antioxidant system, which includes antioxidant enzymes and low-molecular-weight organic compounds that exhibit antioxidant properties [49].

In response to drought, barley plants responded to increasing TBARS levels, indicating an increase in the intensity of membrane lipid peroxidation (Figure 3). Simultaneously, a 3-fold decrease in SOD activity, a 3.2-fold increase in ascorbate peroxidase activity, and a 60% increase in guaiacol-dependent peroxidase activity were observed in barley leaves relative to the control (Figure 4). Pretreatment of plants with HBL slightly (by 20%) stimulated TBARS accumulation, while HCS, on the contrary, relieved the drought-induced increase in TBARS content (Figure 3), i.e., at the level of the lipid peroxidation process, lactone- and ketone-containing BRs had opposite effects on plants under drought conditions. Pretreatment of wheat plants with 24-epibrassinolide followed by PEG6000-induced water deficit also resulted in a reduction in lipid peroxidation and electrolyte leakage from tissues [15]. Pretreatment of barley plants with HCS followed by drought increased SOD activity sevenfold, had no effect on catalase activity, and slightly decreased peroxidase activity relative to water deficit. Compared with drought alone, pretreatment of plants with HBL increased SOD activity threefold, increased catalase activity by 36%, and decreased peroxidase activity (Figure 4). Hence, one of the manifestations of the protective effect of pretreatment of plants with HBL and HCS under subsequent drought conditions is the significant activation of SOD, a key antioxidant defense enzyme. It was previously shown that the treatment of tobacco plants and cut flowers of *Dendrobium* with 24-epibrassinolide under drought conditions resulted in increased resistance by increasing the activities of several antioxidant enzymes [13,20]. According to Yang et al. [37], the dynamics of changes in SOD activity during drought are highly complex. For example, with increasing stress intensity, SOD activity often decreases or remains unchanged. This behavior of antioxidant enzymes, particularly SOD, is clearly explained by the fact that plants respond to tissue water deficit rather than to water deficit per se.

Interestingly, the ability of BRs to increase plant tolerance to drought through the activation of antioxidant enzymes depends on the treatment method. For example, pretreatment of tomato plants with brassinosteroids followed by drought stress resulted in the activation of several antioxidant enzymes, including catalase [50], whereas treatment of plants during stress was accompanied by the inhibition of catalase activity [51].

The nonenzymatic components of antioxidant defense include carotenoids, phenolic compounds, proline, sugars, etc. [49]. It is known, for example, that carotenoids are involved in the quenching of ^1^O_2_ and peroxide, which are generated when chlorophyll is overexcited [52] due to a decrease in the turgor of the closing cells and a reduction, as a consequence, in carbon dioxide entry into the plant. This adaptation strategy aimed at reducing the intensity of oxidative stress by accumulating carotenoids is characteristic mainly of halophytic plants [53]. It is likely that glycophyte barley plants accumulate other antioxidant compounds, which may be the reason for the lack of a stimulating effect of BRs on carotenoid content under water deficit. Other studies have shown that treatment of plants with BRs under drought conditions leads to an increase in soluble sugar content and superoxide dismutase, peroxidase and catalase activities [13,54].

Barley plants responded to drought by intensive (57.9-fold) proline accumulation (Figure 5). This was achieved by stimulating the expression of the key gene for synthesis P5CS1 and simultaneously inhibiting the activity of expression of the key gene for proline degradation PDH (Table 4 and Table S1). Proline, as a “chemical chaperone”, maintains the native conformation of proteins and enzymes such as RUBISCO, exhibits antioxidant properties, and is involved in osmoregulation [27,28,55]. Proline accumulation in drought-stressed plants has been observed many times previously [56,57]. BR treatment of plants is also known to stimulate proline accumulation under water deficit [13,15,20]. At the same time, we showed that pretreatment of barley plants with BRs followed by drought partially reduced stress-induced proline accumulation, despite the fact that in this case there was stimulation of P5CS1 gene expression of proline synthesis and inhibition of its degradation gene expression (Table 4). In this case, proline content was 16.4-fold and 9.5-fold higher in plants pretreated with HBL or HCS followed by drought compared with the control, respectively (Figure 5).

The question arises as to whether the BR inhibition of proline accumulation during water deficit is a unique event. The answer to this question is in the negative. A decrease in proline content in cotton and velvet plants treated with BRs and exposed to drought stress has also been shown previously [58,59]. Evidence is known that BRs reduced salt-stress-induced proline accumulation in rapeseed [24], rice [60], and pepper [61] plants. It is also impossible not to mention the recently published data, according to which exogenous BRs increase drought tolerance in soybean plants; however, it is not involved in the adaptation of plants to water deficit, since the proline level in plants decreases as the BR concentration increases [62]. Interesting results were reported by Cai et al. (2020) [43], according to which an increase in the level of endogenous BRs in rhododendron plants was accompanied by an increase in leaf water potential and a decrease in malonic dialdehyde, proline, and soluble protein. We have shown that pretreatment of barley plants with BRs followed by drought stress resulted in an increase in the content of endogenous steroid phytohormones (Figure 6). This finding makes it more or less likely that endogenous BRs are involved in our observed decrease in hormone-dependent proline accumulation during drought.

Another reason for the BR downregulation of proline accumulation under water deficit may be related to the antioxidant properties exhibited by proline. We are well aware that BR, like other hormones, are specialized metabolic regulators that optimize cellular metabolism, including antioxidant status. Recently, BRs in rice plants have been shown to act as inducers of melatonin synthesis, which is one of the most effective antioxidants [63]. Moreover, barley plants are characterized by a high endogenous content of melatonin and its derivatives [64]. Under these conditions, BRs, by stimulating melatonin synthesis, partially downregulate proline accumulation as an “excess” antioxidant under these conditions, resulting in energy conservation under stress. Moreover, the contribution of proline to cellular osmoregulation is rather low, since its concentration in cytoplasm does not exceed the micromolar level.

### 3.3. Endogenous Contents of Brassinosteroids under Drought and 28-Homobrassinosteroid Pretreatments

Many studies have shown that under drought conditions, there are changes in the levels of endogenous BRs in plants such as pea, soybean, rice, tobacco, maize, and tomato plants (cited by [14]). These changes are characterized by species specificity. Most studies have analyzed either total brassinosteroid content or castasterone and/or brassinolide content. It was previously observed that in the barley cultivar “Bowman” and in plants of five brassinosteroid biosynthesis mutant lines, an increase in castasterone and 24-epibrassinolide content was observed in response to drought [65]. The effect of exogenous BRs on the endogenous content of various groups of steroid phytohormones under stress conditions was first investigated in rape plants under salinity conditions [24]. It should be noted that at present, there are practically no data on the changes in the profile of BRs under drought in response to the treatment of plants with exogenous steroid phytohormones [14].

Our results indicate that barley plants responded to drought not by increasing but, on the contrary, by decreasing the levels of all analyzed BRs—28-homobrassinosteroids, B-lactones, and B-ketones (Figure 6). Pretreatment of barley plants with BRs followed by drought treatment allowed us to show that both HBL and HCS partially restored the content of B-ketones in the aboveground part of barley plants, whereas HCS completely reversed the inhibitory effect of drought on the content of B-lactones (Figure 6). The data obtained are extremely important because they indicate the ability of plant pretreatment with HBL and HCS to partially restore endogenous B-ketone and B-lactone contents during subsequent drought. A number of studies have shown that the level of endogenous BRs largely determines the drought tolerance of plants. In particular, ref. [23] found that the content of BRs in different maize lines correlated with the level of drought tolerance, i.e., highly tolerant genotypes were characterized by the highest content of BRs. Analysis of a brassinosteroid-deficient mutant of cotton showed that it was characterized by increased sensitivity to drought, which is due to the extremely low water-holding capacity of tissues and poor water-absorbing function of the root [32].

## 4. Materials and Methods

### 4.1. Plant Materials and Experimental Design

**Figure 7:**
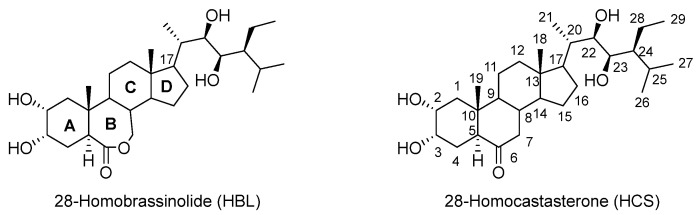
Chemical structure of brassinosteroids.

To study the resistance of monocotyledonous plants to water deficit conditions, barley of the Biom variety was used. Barley seeds were sterilized with 70% ethanol for 5 min, washed with distilled water, and stratified at 4 °C for three days. The seeds were then germinated for two days (in the light, in a phytotron with a 16-h photoperiod at 19–21 °C) on moist filter paper. Germinated seeds were treated for two hours in Petri dishes with 0.1 nM HBL or 0.1 nM HCS while shaking (Figure 7). The control seeds were watered for 2 h under the same conditions as the experimental seeds. These hormone concentrations were selected based on previously obtained results (Table S2) [11]. After the end of the treatment, the seeds were sown 10 at a time into 1-L vessels with soil (Garant soil, Temp-2 soil coarse plant, Tomsk, Russia) according to the following treatments: (1) control, (2) drought, (3) 0.1 nM HBL, (4) 0.1 nM HCS, (5) 0.1 nM HBL + drought, and (6) 0.1 nM HCS + drought. Each variant contained 5 vessels. In the first week of the experiment, all plants were grown under the same soil moisture conditions. Water deficit was created by discontinuing watering a portion of 7-day-old plants for the next two weeks. Plants that did not experience water deficit were watered two days later on the third day. After 2 weeks, the biomass of the aboveground plant parts, water content in the tissues of the aboveground organs, relative water content in the leaves, photosynthetic pigment content, and photochemical activity parameters of PS II were measured. For further analyses, plant samples were fixed in liquid nitrogen and stored at 70 °C.

### 4.2. Synthesis of Brassinosteroids

The synthesis of 28-HBR for these studies was carried out in 12 stages from available stigmasterol according to our previously described method [66].

### 4.3. Determination of Plant Biomass and Water Content

Plant biomasses were determined gravimetrically. For fresh weight estimation, an analytical balance with an accuracy of 1 mg (Sartorius CP 622” (Göttingen, Germany)) was used, whereas for dry weight estimation, an analytical balance with an accuracy of 0.1 mg (AB54-S, Mettler Toledo, Greifensee, Switzerland) was used. In the latter case, the plant samples were predried to a constant weight at 70 °C. The water content is expressed as a percentage of fresh biomass. The relative water content (RWC) was calculated using the following formula [67]: RWC % = (fresh weight − dry weight)/(swollen weight − dry weight) × 100%, where fresh weight is the weight of leaves after cutting them at the base of the lamina. Swollen weight was determined after soaking the leaves in distilled water for 24 h at 60% relative humidity, +4 °C, and low light. The dry weight is the weight of the plant material after drying the leaves at 70 °C for 24 h to a constant weight.

### 4.4. Determining the Osmotic Potential

The osmotic potential of the cell exudates was determined using an Osmomat 030 cryoscopic osmometer (Gonotec, Berlin, Germany) according to the manufacturer’s instructions. Cell sap was squeezed from defrosted leaf samples.

### 4.5. Determination of Chlorophyll Fluorescence

The photochemical activity parameters of PS II were measured using a RAM fluorimeter (MINI-PAM-II, Heinz-Walz, Effeltrich, Germany). The fluorescence coefficients and relative electron transport rates were calculated using MINI-PAM-II software WinControl-3 Software (https://www.walz.com/products/chl_p700/monitoring-pam/wincontrol-3.html accessed on 20 February 2023). The parameters evaluated were Fv/Fm (maximum quantum efficiency of photosystem II, PS II), ETR (electron transport rate), Y(II) (photochemical utilization of excitation energy in photosystem II under active light), qP (photochemical fluorescence quenching coefficient), and NPQ (nonphotochemical fluorescence quenching).

### 4.6. Evaluating Lipid Peroxidation Levels

The level of lipid peroxidation was evaluated by the formation of a stained complex between thiobarbituric acid and thiobarbituric-acid-reactive substances (TBARS) upon heating according to Buege and Aust [68]. The TBARS content was determined spectrophotometrically by estimating the absorbance of the solutions at wavelengths of 532 and 600 nm using a Genesys 10S UV—Vis Genes spectrophotometer (Thermo Fisher Scientific, Waltham, MA, USA).

### 4.7. Determination of Photosynthetic Pigments

The Lichtenthaler method was used to estimate the content of chlorophyll *a* (Chl *a*), chlorophyll *b* (Chl *b*), and carotenoids (Car) [55]. For this purpose, leaf samples (70 mg) were ground in liquid nitrogen, after which the resulting material was transferred to a tube with 96% ethanol (1.5 mL) and calcium carbonate and vigorously vortexed, after which the homogenate was centrifuged for 10 min at 8000× *g* using a MiniSpin centrifuge (Eppendorf, Hamburg, Germany). This extraction was performed three times. The extracts were combined, and the total volume was 5 mL. To estimate the concentration of pigments in the alcoholic extract, the Lichtenthaler formula was used after measuring the optical density of the extract at wavelengths of 470, 648, 664, and 720 nm on a Genesys 10S UV—Vis spectrophotometer [69].

### 4.8. Determination of the Free Proline Content

Free proline was extracted and determined according to Bates et al. [70], and the optical density of the solution was measured at a wavelength of 520 nm on a Genesys 10S UV—Vis Genes spectrophotometer (Thermo Fisher Scientific, USA).

### 4.9. Determination of the Activity of Antioxidant Enzymes

Total superoxide dismutase (EC 1.15.1.1.1), guaiacol-dependent peroxidase (EC 1.11.1.7) (PO), ascorbate-dependent peroxidase (APX; EC 1.11.1.11), and catalase (EC 1.11.1.6) activities were determined in fresh extracts of leaf tissues. Leaf samples (200 mg) were ground in liquid nitrogen with insoluble polyvinyl pyrrolidone, extracted in 0.066 M potassium–phosphate buffer (pH 7.4) containing 0.5 mM dithiothreitol (Sigma-Aldrich, Darmstadt, Germany) and 0.1 mM phenylmethylmethylsulfonyl fluoride in dimethyl sulfoxide (Sigma-Aldrich, Germany) and then centrifuged for 20 min at 8000 rpm and 4 °C using a 5430R centrifuge (Eppendorf, Hamburg, Germany). Total SOD activity was determined according to the methods of Beauchamp and Fridovich [71]. The reaction medium (2 mL) contained 10 µL of supernatant, 1.75 mL of 50 mM Tris-HCl buffer (pH 7.8), 0.2 mL of 0.1 M DL-methionine (Sigma-Aldrich, Germany), 0.063 mL of 1.7 mM nitro blue tetrazolium (Fermentas, Waltham, MA, USA), 0.047 mL of 1% Triton X-100 (Sigma-Aldrich, Germany), and 0.060 mL of 0.004% riboflavin (Sigma-Aldrich, Germany). The reaction proceeded under LED lamps (I = 232 µmol photons/m^−2^ s^−1^) for 30 min. The absorption was measured at 560 nm using a Genesys 10S UV—Vis spectrophotometer (Thermo Fisher Scientific, USA).

Guaiacol-dependent peroxidase activity was determined as previously described [11]. The reaction mixture contained 50 µL of supernatant, 1.95 mL of 0.066 M potassium-phosphate buffer (pH 7.4), 200 µL of 7 mM guaiacol (Sigma-Aldrich, Germany), and 250 µL of 0.01 M H_2_O_2_. The absorption was measured at 470 nm using a Genesys 10S UV—Vis spectrophotometer (Thermo Fisher Scientific, USA). To estimate catalase activity (EC 1.11.1.6) according to the method of Chance and Maehly [72], plant samples were ground in liquid nitrogen with insoluble polyvinylpyrrolidone (Sigma-Aldrich, Germany), extracted with 0.066 M potassium phosphate buffer (pH 7. 8) containing 0.5 M dithiothreitol and 0.1 M phenylmethylsulfonyl fluoride in dimethyl sulfoxide, and then centrifuged for 20 min at 10,000 rpm and 4 °C (Eppendorf 5430R, Hamburg, Germany). The reaction mixture contained 50 μL of supernatant, 1.9 mL of 0.066 M potassium phosphate buffer (pH 7.8), and 50 μL of 0.05 M H_2_O_2_. The absorbance of the solution was measured at 240 nm on a spectrophotometer (Genesys 10S UV—Vis, ThermoScientific, USA).

For the determination of ascorbate peroxidase (AP, KF 1.11.1.11.) activity, fresh plant material was homogenized at 4 °C in 0.05 M Tris-HCl1 buffer (pH 7.8) containing 1 mM EDTA, 1 mM ascorbic acid (AA), 5% (weight/weight) polyvinylpyrrolidone (PVP) (Dia-M, Moscow, Russia), and 10% (weight/volume) sorbitol (Sigma-Aldrich, Germany). The resulting extract was filtered through 2 layers of Capron cloth and centrifuged at 12,000× *g* for 30 min. The activity of ascorbate peroxidase was determined spectrophotometrically by the decrease in light absorption at 290 nm caused by the oxidation of ascorbate. The reaction mixture (1 mL) contained 0.06 M sodium phosphate buffer (pH 7.0), 0.1 mM EDTA (Sigma-Aldrich, Germany), 0.5 mM AA, 100 μL of extract, and 0.1 mM H_2_O_2_ [73,74].

### 4.10. Determination of Total Protein Content

The Esen method was used to determine the protein content of the plant material [75].

### 4.11. Determination of the Content of Endogenous Brassinosteroids

The contents of B-lactones, B-ketones, and 28-homobrassinosteroids were determined with previously developed test systems [76] using a two-stage method [77]. A detailed description of the method used for the analysis of BRs is presented in the Supplementary Materials.

### 4.12. Selection of Target Genes for qRT—PCR Analysis and Primer Design

*The* PCR conditions and primer sequences for the reference genes were obtained from Nicot et al. [78]. The primary structures of the proline synthesis and degradation genes (P5CS1, P5CR, PDH, and P5CDH) were obtained from the NCBI database (http://www.ncbi.nlm.nih.gov/, accessed on 20 February 2023). The primer-BLAST [79] and Vector NTI 11.0 programs were used to design the gene-specific primers. The inventory numbers of the target genes, primer sequences, and PCR product sizes are presented in the Supplementary Materials (Table S3).

### 4.13. Quantitative RT—PCR Analysis of Target Gene Expression

Leaf samples were ground in liquid nitrogen, and RNA was extracted with the method proposed by Bilgin D. [80]. Samples were treated with RNase-free DNAse I (Thermo Fisher Scientific, USA) to remove genomic DNA. MMLV-RevertAid reverse transcriptase (Evrogen, Moscow, Russia) was used for cDNA synthesis.

Gene transcript levels were determined by real-time PCR on a LightCycler^®^ 96 system (Roche, Switzerland) using SYBR Green I (Evrogen, Russia). The reaction mixture contained the HS SYBR qPCR mix (Evrogen, Russia), cDNA, PCR primers (Evrogen, Russia), and ddH_2_O. Three technical replicates of each sample were treated, and ddH_2_O was used as a negative control. The amplification conditions were as follows: 95 °C for 5 min, followed by 40 cycles of 95 °C for 15 s, Tm°C (Table S3) for 30 s, and 72 °C for 15 s. The transcript levels of target genes were normalized to ARF1 gene expression. Transcript levels were calculated using MS Excel according to the manufacturer’s instructions.

### 4.14. Statistical Analysis

The experiments were conducted at least three times. One biological replicate included 9 to 12 plants. The values are expressed as mean ± SD. The results were evaluated using one-way analysis of variance (ANOVA) followed by Duncan’s multiple range test (DMRT), using the SPSS 26 software package. *p*-values less than 0.05 were regarded as statistically significant.

## 5. Conclusions

Drought is one of the leading negative factors of global climate change. It reduces the yield of the most important crops, which makes it urgent to develop methods to reduce the negative effects of water deficit on plants. BRs are a class of steroidal phytohormones that regulate plant growth, development, and defense responses to abiotic stresses, including drought. To date, virtually no research has investigated the stress-protective effects of BRs with different chemical structures. In this work, we studied the ability of HBL and HCS to increase the drought tolerance of barley *(Hordeum vulgare* L.) plants and to alter the endogenous status of BRs. Germinated barley seeds were treated with solutions of 0.1 nM (HBL) and 0.1 nM (HCS) for two hours. A water deficit was created by stopping the watering of 7-day-old plants for the next two weeks. Plants respond to drought through growth inhibition, water disturbance, increased lipid peroxidation, and differential effects on antioxidant enzyme activities, intense proline accumulation, altered expression of genes involved in metabolism, and decreased levels of endogenous hormones (B-ketones BRs and B-lactones BRs). Pretreatment of barley with steroidal phytohormones of different chemical structures increased plant resistance to subsequent drought, which was manifested in a partial reduction in the inhibitory effect of water deficit on the accumulation of fresh biomass in the case of HCS and fresh and dry biomass in the case of HBL. Moreover, HCS increased the osmotic potential, which may be explained by the partial suppression of proline accumulation and a decrease in the intensity of lipid peroxidation. The latter was manifested by a sevenfold increase in SOD activity compared with the effect of drought alone. Short-term pretreatment of barley seedlings with HBL increased SOD activity and catalase activity threefold (by 36%) under water deficit conditions. At the same time, the hormones did not affect the functional activity of PSII but partially restored the endogenous content of B-ketones and B-lactones, which often correlates with an increase in plant resistance. Demonstration of the protective effect of lactone- and ketone-brassinosteroids of the 28-homobrassinolide series on barley plants under water deficit may attract the interest of researchers to this problem and expand the range of practically relevant brassinosteroids.

## Figures and Tables

**Table 1:** Effect of drought and plant pretreatment with 28-homobrassinolide (HBL) and 28-homocastasterone (HCS) on biomass accumulation and relative water content of barley plants.

Treatment	Fresh Biomass		Dry Biomass		Relative Water Content
	g	%	g	%	
Control	0.505 ± 0.016 ^a^	100	0.042 ± 0.002 ^ac^	100	1.0 ± 0.0 ^a^
Drought	0.286 ± 0.014 ^b^	57	0.032 ± 0.003 ^b^	76	0.8 ± 0.1 ^b^
HBL	0.488 ± 0.021 ^a^	97	0.041 ± 0.004 ^ac^	99	1.0 ± 0.0 ^a^
HCS	0.545 ± 0.017 ^a^	108	0.049 ± 0.003 ^a^	118	0.9 ± 0.1 ^ac^
HBL + drought	0.363 ± 0.012 ^c^	72	0.039 ± 0.002 ^c^	94	0.9 ± 0.0 ^cd^
HCS + drought	0.325 ± 0.011 ^d^	64	0.031 ± 0.003 ^b^	73	0.9 ± 0.0 ^bd^

Values are given as the mean ± SD for each treatment. Values not sharing a common or same alphabet letter (^a–d^), and they differ significantly at *p* < 0.05 (Duncan’s multiple range test).

**Table 2:** Effect of HBL, HCS, and drought on the content of photosynthetic pigments in leaves of barley plants.

Treatment	Chlorophyll *a* Content		Chlorophyll *b* Content		Carotenoids Content	
	mg/dry Weight	%	mg/dry Weight	%	mg/dry Weight	%
Control	13.20 ± 0.36 ^a^	100	3.33 ± 0.11 ^a^	100	4.82 ± 0.17 ^a^	100
Drought	9.28 ± 0.54 ^b^	70	2.39 ± 0.15 ^b^	72	3.11 ± 0.16 ^b^	65
HBL	12.14 ± 0.29 ^c^	92	3.08 ± 0.10 ^a^	93	4.49 ± 0.26 ^a^	93
HCS	12.75 ± 0.48 ^ac^	97	3.25 ± 0.21 ^a^	98	4.81 ± 0.37 ^a^	100
HBL + drought	7.96 ± 0.33 ^d^	60	1.97 ± 0.09 ^c^	59	2.68 ± 0.12 ^c^	56
HCS + drought	10.40 ± 0.29 ^e^	79	2.69 ± 0.09 ^b^	81	3.40 ± 0.30 ^b^	70

Values are given as the mean ± SD for each treatment. Values not sharing a common or same alphabet letter (^a–e^), and they differ significantly at *p* < 0.05 (Duncan’s multiple range test).

**Table 3:** Effects of HBL, HCS, and drought on chlorophyll *a* fluorescence parameters in the leaves of barley plants.

Treatment	Chlorophyll *a* Fluorescence Parameters				
	Y(II)	ETR	qP	NPQ	Fv/Fm
Control	0.66 ± 0.00 ^a^	24.98 ± 0.21 ^a^	0.79 ± 0.02 ^a^	0.83 ± 0.03 ^a^	0.81 ± 0.01 ^a^
%	100	100	100	100	100
Drought	0.66 ± 0.02 ^a^	24.08 ± 1.21 ^a^	0.75 ± 0.04 ^a^	0.82 ± 0.03 ^a^	0.83 ± 0.00 ^a^
%	100	96	95	99	102
HBL	0.66 ± 0.00 ^a^	25.01 ± 0.15 ^a^	0.83 ± 0.02 ^a^	0.77 ± 0.04 ^a^	0.83 ± 0.01 ^a^
%	100	100	105	93	101
HCS	0.67 ± 0.01 ^a^	25.26 ± 0.28 ^a^	0.80 ± 0.03 ^a^	0.84 ± 0.05 ^a^	0.83 ± 0.01 ^a^
%	101	101	101	101	102
HBL + drought	0.67 ± 0.01 ^a^	25.47 ± 0.41 ^a^	0.76 ± 0.02 ^a^	0.78 ± 0.07 ^a^	0.82 ± 0.00 ^a^
%	102	102	96	93	101
HCS + drought	0.66 ± 0.01 ^a^	25.40 ± 0.46 ^a^	0.73 ± 0.02 ^b^	0.76 ± 0.04 ^a^	0.83 ± 0.01 ^a^
%	100	102	93	91	102

Values are given as the mean ± SD for each treatment. Values not sharing a common or same alphabet letter (^a,b^), and they differ significantly at *p* < 0.05 (Duncan’s multiple range test).

**Table 4:** The effects of drought and pretreatment with brassinosteroids on the expression of genes involved in proline metabolism in shoots of barley plants.

	*P5CS1*	*P5CR*	*PDH*	*P5CDH*
Control	1.00 *^a^*	1.00 *^a^*	1.00 *^a^*	1.00 *^a^*
Drought	4.38 *^b^*	2.51 *^b^*	0.34 *^b^*	4.96 *^b^*
HBL	2.06 *^c^*	1.23 *^ac^*	0.64 *^a^*	3.12 *^c^*
HCS	3.84 *^b^*	1.41 *^c^*	1.05 *^a^*	2.27 *^d^*
HBL + drought	3.43 *^bc^*	1.50 *^c^*	0.24 *^b^*	0.94 *^a^*
HCS + drought	2.68 *^c^*	3.01 *^d^*	0.37 *^b^*	2.93 *^d^*

The value in the control plants was taken as 1.0. Values are given as the mean ± SD for each treatment. Values not sharing a common or same alphabet letter (*^a^*^–*d*^), and they differ significantly at *p* < 0.05 (Duncan’s multiple range test).

## Data Availability

The original contributions presented in the study are included in the article; further inquiries can be directed to the corresponding author.

## Supplementary Materials

The following supporting information can be downloaded at https://www.mdpi.com/article/10.3390/plants13101345/s1, Table S1: Transcript levels of proline metabolism genes; Table S2: Effect of different concentrations of BS on accumulation of fresh and dry biomass of barley plants under normal conditions and under water deficit; Table S3: List of genes, gene-specific primers, and PCR conditions; analysis of endogenous BRs level.

## References

[1] Zhang H., Zhu J., Gong Z., Zhu J.K. (2022). Abiotic stress responses in plants. Nat. Rev. Genet..

[2] Ghadirnezhad Shiade S.R., Fathi A., Taghavi Ghasemkheili F., Amiri E., Pessarakli M. (2023). Plants’ responses under drought stress conditions: Effects of strategic management approaches—A review. J. Plant Nutr..

[3] The High Cost of Drought. https://www.drought.gov/news/high-cost-drought/.

[4] Cáceres-Cevallos G.J., Albacete-Moreno A.A., Ferreres F., Gil-Izquierdo Á., Jordán M.J. (2023). Evaluation of the physiological parameters in *Lavandula latifolia* Medik. under water deficit for preselection of elite drought-resistant plants. Ind. Crops Prod..

[5] Kumar S., Bhushan B., Wakchaure G.C., Meena K.K., Kumar M., Meena N.L., Rane J., Lone R., Shuab R., Kamili A.N. (2020). Plant Phenolics Under Water-Deficit Conditions: Biosynthesis, Accumulation, and Physiological Roles in Water Stress Alleviation. Plant Phenolics in Sustainable Agriculture: Volume 1.

[6] Ibrahim M.F.M., Ibrahim H.A., Abd El-Gawad H.G. (2021). Folic acid as a protective agent in snap bean plants under water deficit conditions. J. Hortic. Sci. Biotechnol..

[7] Shafiq S., Akram N.A., Ashraf M., García-Caparrós P., Ali O.M., Latef A. (2021). Influence of glycine betaine (natural and synthetic) on growth, metabolism and yield production of drought-stressed maize (*Zea mays* L.) plants. Plants.

[8] Elkelish A., El-Mogy M.M., Niedbała G., Piekutowska M., Atia M.A.M., Hamada M.M.A., Shahin M., Mukherjee S., El-Yazied A.A., Shebl M. (2021). Roles of exogenous α-lipoic acid and cysteine in mitigation of drought stress and restoration of grain quality in wheat. Plants.

[9] Salvi P., Manna M., Kaur H., Thakur T., Gandass N., Bhatt D., Muthamilarasan M. (2021). Phytohormone signaling and crosstalk in regulating drought stress response in plants. Plant Cell Rep..

[10] Efimova M.V., Ahammed G.J., Sharma A., Yu J. (2022). Application of brassinosteroids for improving crop production: From laboratory to field. Brassinosteroids in Plant Developmental Biology and Stress Tolerance.

[11] Kolomeichuk L.V., Efimova M.V., Zlobin I.E., Kreslavski V.D., Murgan O.K., Kovtun I.S., Khripach V.A., Kuznetsov V.V., Allakhverdiev S.I. (2020). 24-Epibrassinolide alleviates the toxic effects of NaCl on photosynthetic processes in potato plants. Photosynth. Res..

[12] Zlobin I.E., Danilova E.D., Murgan O.K., Kolomeichuk L.V., Litvinovskaya R.P., Sauchuk A.L., Kuznetsov V.V., Efimova M.V. (2023). Structurally different exogenic brassinosteroids protect plants under polymetallic pollution via structure-specific changes in metabolism and balance of cell-protective components. Molecules.

[13] Khan R., Ma X., Hussain Q., Asim M., Iqbal A., Ren X., Shah S., Chen K., Shi Y. (2022). Application of 2,4-epibrassinolide improves drought tolerance in tobacco through physiological and biochemical mechanisms. Biology.

[14] Marková H., Tarkowská D., Čečetka P., Kočová M., Rothová O., Holá D. (2023). Contents of endogenous brassinosteroids and the response to drought and/or exogenously applied 24-epibrassinolide in two different maize leaves. Front. Plant Sci..

[15] Avalbaev A., Fedyaev V., Lubyanova A., Yuldashev R., Allagulova C. (2024). 24-Epibrassinolide reduces drought-induced oxidative stress by modulating the antioxidant system and respiration in wheat seedlings. Plants.

[16] Sarfraz M., Hussain S., Ijaz M., Nawaz A., Yasir T.A., Sher A., Wasaya A., Ahmad S., Hasanuzzaman M., Fotopoulos V. (2019). Abiotic Stress Tolerance in Plants by Priming and Pretreatments with Phytohormones. Priming and Pretreatment of Seeds and Seedlings: Implication in Plant Stress Tolerance and Enhancing Productivity in Crop Plants.

[17] Rhaman M.S., Imran S., Rauf F., Khatun M., Baskin C.C., Murata Y., Hasanuzzaman M. (2020). Seed priming with phytohormones: An effective approach for the mitigation of abiotic stress. Plants.

[18] Efimova M.V., Kolomeichuk L.V., Boyko E.V., Malofii M.K., Vidershpan A.N., Plyusnin I.N., Golovatskaya I.F., Murgan O.K., Kuznetsov V.V. (2018). Physiological mechanisms of *Solanum tuberosum* L. plants’ tolerance to chloride salinity. Russ. J. Plant Physiol..

[19] Hudeček M., Nožková V., Plíhalová L., Plíhal O. (2022). Plant hormone cytokinin at the crossroads of stress priming and control of photosynthesis. Front. Plant Sci..

[20] Sukpitak C., Munné-Bosch S., Seraypheap K. (2024). Brassinosteroids alleviate postharvest water deficit stress in cut *Dendrobium* K‘hao Sanan’ orchid through modulation of protective mechanisms, osmotic regulation, and endogenous hormonal levels. Postharvest Biol. Technol..

[21] Kolomeichuk L.V., Danilova E.D., Khripach V.A., Zhabinskii V.N., Kuznetsov V.V., Efimova M.V. (2021). Ability of lactone- and ketone-containing brassinosteroids to induce priming in rapeseed plants to salt stress. Russ. J. Plant Physiol..

[22] Zhang W., Huang H., Zhou Y., Zhu K., Wu Y., Xu Y., Wang W., Zhang H., Gu J., Xiong F. (2023). Brassinosteroids mediate moderate soil-drying to alleviate spikelet degeneration under high temperature during meiosis of rice. Plant Cell Environ..

[23] Gillani S.F.A., Zhuang Z., Rasheed A., Haq I.U., Abbasi A., Ahmed S., Wang Y., Khan M.T., Sardar R., Peng Y. (2022). Brassinosteroids induced drought resistance of contrasting drought-responsive genotypes of maize at physiological and transcriptomic levels. Front. Plant Sci..

[24] Efimova M.V., Savchuk A.L., Hasan J.A.K., Litvinovskaya R.P., Khripach V.A., Kholodova V.P., Kuznetsov V.V. (2014). Physiological mechanisms of enhancing salt tolerance of oilseed rape plants with brassinosteroids. Russ. J. Plant Physiol..

[25] Efimova M.V., Khripach V.A., Boyko E.V., Malofii M.K., Kolomeichuk L.V., Murgan O.K., Vidershpun A.N., Mukhamatdinova E.A., Kuznetsov V.V. (2018). The priming of potato plants induced by brassinosteroids reduces oxidative stress and increases salt tolerance. Dokl. Biol. Sci..

[26] Tatsiana G.K., Nikolay V.K. (2020). The effect of soil drought on the content of photosynthetic pigments in barley plants of the Brovar variety. Exp. Biol. Biotechnol..

[27] Kawagoe S., Nakagawa H., Kumeta H., Ishimori K., Saio T. (2018). Structural insight into proline cis/trans isomerization of unfolded proteins catalyzed by the trigger factor chaperone. J. Biol. Chem..

[28] Ghosh U.K., Islam M.N., Siddiqui M.N., Cao X., Khan M.A.R. (2022). Proline, a multifaceted signalling molecule in plant responses to abiotic stress: Understanding the physiological mechanisms. Plant Biol..

[29] Miao R., Li C., Liu Z., Zhou X., Chen S., Zhang D., Luo J., Tang W., Wang C., Wu J. (2024). The role of endogenous brassinosteroids in the mechanisms regulating plant reactions to various abiotic stresses. Agronomy.

[30] Farooq M., Wahid A., Basra S.M.A., Islam-ud-Din. (2009). Improving water relations and gas exchange with brassinosteroids in rice under drought stress. J. Agron. Crop Sci..

[31] Khodyankov A.A. (2008). The influence of brassinosteroids on the resistance of fiber flax plants to drought. Agrochem. Bull..

[32] Chen E., Zhang X., Yang Z., Zhang C., Wang X., Ge X., Li F. (2019). BR deficiency causes increased sensitivity to drought and yield penalty in cotton. BMC Plant Biol..

[33] Souza A.C.D.d., Reis B.S.S., Bacin D.F., Oliveira L.R.d., Santos W.S.d., Borges L.P., Cardoso N.C., Matos F.S. (2023). Importance of brassinosteroids for mitigating water stress in sorghum. Aust. J. Crop Sci..

[34] Murchie E.H., Lawson T. (2013). Chlorophyll fluorescence analysis: A guide to good practice and understanding some new applications. J. Exp. Bot..

[35] Baker N.R., Rosenqvist E. (2004). Applications of chlorophyll fluorescence can improve crop production strategies: An examination of future possibilities. J. Exp. Bot..

[36] Robredo A., Pérez-López U., Lacuesta M., Mena-Petite A., Muñoz-Rueda A. (2010). Influence of water stress on photosynthetic characteristics in barley plants under ambient and elevated CO_2_ concentrations. Biol. Plant.

[37] Yang X., Lu M., Wang Y., Wang Y., Liu Z., Chen S. (2021). Response mechanism of plants to drought stress. Horticulturae.

[38] Kalaji H.M., Carpentier R., Allakhverdiev S.I., Bosa K. (2012). Fluorescence parameters as early indicators of light stress in barley. J. Photochem. Photobiol. B.

[39] Janeczko A., Oklešťková J., Pociecha E., Kościelniak J., Mirek M. (2011). Physiological effects and transport of 24-epibrassinolide in heat-stressed barley. Acta Physiol. Plant..

[40] Simkin A.J., Kapoor L., Doss C.G.P., Hofmann T.A., Lawson T., Ramamoorthy S. (2022). The role of photosynthesis related pigments in light harvesting, photoprotection and enhancement of photosynthetic yield in planta. Photosynth. Res..

[41] Stefanov M., Rashkov G., Borisova P., Apostolova E. (2023). Sensitivity of the photosynthetic apparatus in maize and sorghum under different drought levels. Plants.

[42] Tang C.-J., Luo M.-Z., Zhang S., Jia G.-Q., Tang S., Jia Y.-C., Zhi H., Diao X.-M. (2023). Variations in chlorophyll content, stomatal conductance and photosynthesis in Setaria EMS mutants. J. Integr. Agric..

[43] Cai Y.-F., Peng L.-F., Li S.-F., Zhang L., Xie W.-J., Song J., Wang J.-H. (2020). 24-epibrassionlide improves photosynthetic response of *Rhododendron delavayi* to drought. Nord. J. Bot..

[44] Sonjaroon W., Jutamanee K., Khamsuk O., Thussagunpanit J., Kaveeta L., Suksamrarn A. (2018). Impact of brassinosteroid mimic on photosynthesis, carbohydrate content and rice seed set at reproductive stage under heat stress. ANRES.

[45] Hu W.-H., Yan X.H., Xiao Y.A., Zeng J.J., Qi H.J., Ogweno J.O. (2013). 24-Epibrassinosteroid alleviate drought-induced inhibition of photosynthesis in *Capsicum annuum*. Sci. Hortic..

[46] Junior U.O.B., Lima M.D.R., Alsahi A.A., Lobato A.K.S. (2020). Unraveling the roles of brassinosteroids in alleviating drought stress in young Eucalyptus urophylla plants: Implications on redox homeostasis and photosynthetic apparatus. Physiol. Plant..

[47] Qi J., Song C.P., Wang B., Zhou J., Kangasjärvi J., Zhu J.K., Gong Z. (2018). Reactive oxygen species signaling and stomatal movement in plant responses to drought stress and pathogen attack. J. Integr. Plant Biol..

[48] Farooq M.A., Niazi A.K., Akhtar J., Saifullah, Farooq M., Souri Z., Karimi N., Rengel Z. (2019). Acquiring control: The evolution of ROS-Induced oxidative stress and redox signaling pathways in plant stress responses. Plant Physiol. Biochem..

[49] Bose J., Rodrigo-Moreno A., Shabala S. (2014). ROS homeostasis in halophytes in the context of salinity stress tolerance. J. Exp. Bot..

[50] Ziaie J., Kalantari K.M., Behnamnia M. (2009). The effects of brassinosteroid on the induction of biochemical changes in *Lycopersicon esculentum* under drought stress. Turk. J. Bot..

[51] Sivakumar R., Nandhitha G.K., Chandrasekaran P., Boominathan P., Senthilkumar M. (2017). Impact of pink pigmented facultative methylotroph and PGRs on water status, photosynthesis, proline and NR activity in tomato under drought. Int. J. Curr. Microbiol. App. Sci..

[52] Demmig-Adams B., Adams W.W. (1996). The role of xanthophyll cycle carotenoids in the protection of photosynthesis. Trends Plant Sci..

[53] Ozgur R., Uzilday B., Sekmen A.H., Turkan I. (2013). Reactive oxygen species regulation and antioxidant defence in halophytes. Funct. Plant Biol..

[54] Ahmad Lone W., Majeed N., Yaqoob U., John R. (2022). Exogenous brassinosteroid and jasmonic acid improve drought tolerance in *Brassica rapa* L. genotypes by modulating osmolytes, antioxidants and photosynthetic system. Plant Cell Rep..

[55] Kuznetsov V.V., Shevyakova N.I. (1999). Proline under stress: Biological role, metabolism, and regulation. Russ. J. Plant Physiol..

[56] Furlan A.L., Bianucci E., Giordano W., Castro S., Becker D.F. (2020). Proline metabolic dynamics and implications in drought tolerance of peanut plants. Plant Physiol. Biochem..

[57] Samad R., Begum H.H., Saha S., Nasrin S. (2020). Impacts of drought stress on growth, protein, proline, pigment content and antioxidant enzyme activities in rice (*Oryza sativa* L. var. BRRI dhan-24). Dhaka Univ. J. Biol. Sci..

[58] Hanafy Ahmed A.H., Darwish E., Alobaidy M.G. (2017). Impact of putrescine and 24-epibrassinolide on growth, yield and chemical constituents of cotton (*Gossypium barbadense* L.) plant grown under drought stress conditions. Asian J. Plant Sci..

[59] Hemmati K., Ebadi A., Khomari S., Sedghi M. (2018). Influence of ascorbic acid and 24-epibrassinolide on physiological characteristics of pot marigold under water-stress condition. J. Plant Interact..

[60] Ozdemir F., Bor M., Demiral T., Turkan I. (2004). Effects of 24-epibrassinolide on seed germination, seedling growth, lipid peroxidation, proline content and antioxidative system of rice (*Oryza sativa* L.) under salinity stress. Plant Growth Regul..

[61] Maaouia S.I., Denden M., Mouhandes B.D. (2010). Effects of 24-epibrassinolide on growth, chlorophyll, electrolyte leakage and proline by pepper plants under NaCl-stress. Eurasian J. Biosci..

[62] Phan V.H., Le T.T.H., Pham D.M., Nguyen L.T.T., Nguyen K.C., Bui T.M. (2024). Effects of concentration and time of brassinosteroid treatment on growth and yield od soybean under drought stress conditions. Plant Stress Today.

[63] Hwang O.J., Back K. (2022). Molecular Regulation of Antioxidant Melatonin Biosynthesis by Brassinosteroid Acting as an Endogenous Elicitor of Melatonin Induction in Rice Seedlings. Antioxidants.

[64] Yang X., Chen J., Ma Y., Huang M., Qiu T., Bian H., Han N., Wang J. (2022). Function, mechanism, and application of plant melatonin: An update with a focus on the cereal crop, barley (*Hordeum vulgare* L.). Antioxidants.

[65] Gruszka D., Janeczko A., Dziurka M., Pociecha E., Oklestkova J., Szarejko I. (2016). Barley brassinosteroid mutants provide an insight into phytohormonal homeostasis in plant reaction to drought stress. Front. Plant Sci..

[66] Khripach V.A., Litvinovskaya R.P., Raiman M.E., Drach C.V., Zhabinskii V.N., Sviridov O.V., Pryadko A.G., Novick T.V. (2008). Synthesis and immunochemical determination of 28-homobrassinosteroids. Vestsi Natl. Acad. Sci. Belarus Ser. Khim. Sci..

[67] Schonfeld M.A., Johnson R.C., Carver B.F., Mornhinweg D.W. (1988). Water relations in winter wheat as drought resistance indicators. Crop Sci..

[68] Buege J.A., Aust S.D. (1978). Microsomal lipid peroxidation. Methods Enzymol..

[69] Lichtenthaler H.K. (1987). Chlorophylls and carotenoids: Pigments of photosynthetic biomembranes. Methods Enzymol..

[70] Bates L.S., Waldren R.P., Teare I.D. (1973). Rapid determination of free proline for water-stress studies. Plant Soil.

[71] Chance B., Maehly A.C. (1955). Assay of catalases and peroxidases. Methods Enzymol..

[72] Beauchamp C., Fridovich I. (1971). Superoxide dismutase: Improved assays and an assay applicable to acrylamide gels. Anal. Biochem..

[73] Nakano Y., Asada K. (1981). Hydrogen peroxide is scavenged by ascorbate-specific peroxidase in spinach chloroplasts. Plant Cell Physiol..

[74] Gerbling K.P., Kelly G.J., Fischer K.H., Latzko E. (1984). Partial purification and properties of soluble ascorbate peroxidases from pea leaves. J. Plant Physiol..

[75] Esen A. (1978). A simple method for quantitative, semiquantitative, and qualitative assay of protein. Anal. Biochem..

[76] Khripach V.A., Zhabinskii V.N., Litvinovskaya R.P., Hayat S., Ahmad A. (2011). Immunoassays of Brassinosteroids. Brassinosteroids: A Class of Plant Hormone.

[77] Pradko A.G., Litvinovskaya R.P., Sauchuk A.L., Drach S.V., Baranovsky A.V., Zhabinskii V.N., Mirantsova T.V., Khripach V.A. (2015). A new ELISA for quantification of brassinosteroids in plants. Steroids.

[78] Nicot N., Hausman J.F., Hoffmann L., Evers D. (2005). Housekeeping gene selection for real-time RT-PCR normalization in potato during biotic and abiotic stress. J. Exp. Bot..

[79] Primer-BLAST. https://www.ncbi.nlm.nih.gov/tools/primer-blast/.

[80] Bilgin D.D., DeLucia E.H., Clough S.J. (2009). A robust plant RNA isolation method suitable for Affymetrix GeneChip analysis and quantitative real-time RT-PCR. Nat. Protoc..

