# Supplementary material for: Effects of Lactone- and Ketone-Brassinosteroids of the 28-Homobrassinolide Series on Barley Plants under Water Deficit
Source: Plants (Basel). 2024 May 13;13(10):1345. doi: 10.3390/plants13101345 (PMC11124923; doi:10.3390/plants13101345)
Supplement: Supplementary file 1 [file plants-13-01345-s001.zip › plants-2922813-supplementary.pdf]

Supplementary Materials

Table S1. Transcript levels of proline metabolism genes, % of control

Treatments	***P5CS1***	***P5CR***	***PDH***	***P5CDH***
Control	100±18 ^{a}	100±4 ^{a}	100±11 ^{a}	100±24 ^{a}
drought	438±38 ^{b}	251±9 ^{b}	34±12 ^{b}	496±18 ^{b}
HBL	206±27 ^{c}	123±11 ^{ac}	64±12 ^{a}	312±23 ^{c}
HCS	384±70 ^{b}	141±10 ^{c}	105±27 ^{a}	227±66 ^{d}
HBL+drought	343±41 ^{bc}	150±16 ^{c}	24±4 ^{b}	94±48 ^{a}
HCS+drought	268±12 ^{c}	301±13 ^{d}	37±0 ^{b}	293±78 ^{d}

The value in the control plants was taken as 1.0. The values are presented as the means ± standard deviations. Values not sharing a common or same alphabet letter (a-d) and they differ significantly at $p < 0.05$ (Duncan's multiple range test).

Table S2. Effect of different concentrations of BS on accumulation of fresh and dry biomass of barley plants under normal conditions and under water deficit

**Treatment**		**Fresh biomass**		**Dry biomass**	
		**g**	**%**	**g**	**%**
Control		0.470±0.012 [a]	100	0,049±0.003 [a]	100
Drought	0 nM BRs	0.225±0.015 [b]	47	0,031±0.002 [b]	63
	0.001 nM HBL	0.335±0.018 [c]	71	0,038±0.003 [bc]	77
	0.1 nM HBL	0.420±0.021 [d]	89	0,044±0.003 [a]	90
	10 nM HBL	0.214±0.009 [b]	45	0.035±0.002 [b]	71
	0.001 nM HCS	0.195±0.015 [b]	41	0.025±0.002 [d]	51
	0.1 nM HCS	0.334±0.020 [c]	71	0.040±0.002 [c]	81
	10 nM HCS	0.301±0.018 [c]	64	0.029±0.001 [b]	59

The values are presented as the means ± standard deviations. Values not sharing a common or same alphabet letter (a-d) and they differ significantly at $p < 0.05$ (Duncan's multiple range test).

Table S3. List of genes, gene-specific primers and PCR conditions

Gene ID	Gene	F (5'→ 3')	R (5'→ 3')	Tm, °C	Amplicon size for cDNA/DNA, bp
LOC123445639	*P5CS1*, the gene encoding the enzyme delta1-pyrroline-5-carboxylate synthase 1	CCATCAGCACGAGGAAGG	TCAGTTCCAATGCCAGTAGACC	62	90/284
LOC123445483	*P5CR,* the gene encoding the enzyme pyrroline-5-carboxylate reductase	AGAGACGGCTACTGAGAATGATGAA	AGCAACTCCACCATCAGCCA	62	169/267
LOC123438304	*PDH2,* the gene encoding the enzyme proline dehydrogenase 2	CCTCGGCGTCAAGATCGTCC	TTGTAGCAGTCGTGGGTGTCCT	62	117
HORVU.MOREX.r3.1HG0081000 *LOC123448360*	*P5CDH,* the gene encoding the enzyme delta-1-pyrroline-5-carboxylate dehydrogenase 12A1	GCCTGCTAACTGGAACTGGATA	GCTCTAAGTGGGTTGTGAAGTC	62	144/299
LOC123444675	*ADP,* the gene encoding the ribozylation factor 1	CGTGACGCTGTGTTGCTTGT	CCGCATTCATCGCATTAGG	62	61

Analysis of endogenous BS level

The endogenous BS was extracted and quantitated as follows. Samples of barley leaves (3-5 g fresh weight) were weighed, frozen and the frozen leaves were freeze-dried. Lyophilized samples of the barley leaves were pulverized, and the alcohol soluble compounds were extracted with methanol (15 mL) for 24 h at 21±2°C).

The residue was filtered using a glass filter and washed twice with methanol (2 × 10 mL). The methanolic extract was evaporated to dryness, and the residue was partitioned between cyclohexane (7 mL) and 80% aqueous methanol (3 × 4 mL). The combined water-methanol fraction was evaporated to dryness; the residue was dissolved in 1 mL methanol, and then applied to a preparative silica gel plate (20 × 20 cm). A chloroform:methanol (88:12) mixture was used as the eluent. The gel area with a retardation factor (Rf) of 0.4-0.6 was sampled, transferred to a filter, eluted with methanol, and the solvent was evaporated. The resulting residue was dissolved in 2 mL of buffer (0.05 M Tris–hydrochloride [HCl], pH 7.4, containing 0.9% sodium chloride [NaCl], 0.1% bovine serum albumin [BSA], and 0.02% Tween 20). The buffer extract was centrifuged (13 000 g for 10 min), the supernatant was diluted 2-4-fold and used for the BS analysis.

Three earlier developed enzyme immunoassay test systems were used for the quantitation of BS in the plant material [30, 31]. The antibodies of the first test system specifically bind to steroids containing 2_,3_-diol,22*R*,23*R*-diol and 24*R*-methyl groups (EBRs) [32] while those of the second test system specifically bind to steroids containing 2_,3_-diol,22*R*,23*R*-diol and 24*S*ethyl groups (HBRs) [33]. In addition, the antibodies of the third test system specifically bind to steroids containing 2_,3_-diol,22*R*,23*R*-diol and 24*S*-methyl groups (BRs) [34].

Aliquots (50 µL) of the standard (0, 1, 3, 10, 30 and 100 nM) and experimental samples, were analyzed in duplicate by pipetting them into polystyrene wells with immobilized antibodies, followed by the addition of 100 µL of an appropriate BS and horseradish peroxidase-conjugated (HRP) solutions and incubation for 1 h at 37°C. The wells were washed with an appropriate solution (4 × 150 µL), 100 µL chromogenic substrate buffer was added, and the system was incubated for 15 min at 37°C. The reaction was terminated by adding 50 µL 5% sulphuric acid (H_2SO_4). The optical density of the solution in each well was measured at 450 nm and final values were calculated by interpolation from the calibration curve. The dependence of B/B0•100% on the brassinosteroid concentration in the calibration standards (nmol L^{-1}) was plotted in the logit-log coordinates; here B and B0 are optical density values of enzymatic reaction product in the presence of free brassinosteroid (in conditions of the competitive binding of the native hormone and the labeled hormone with antibody) and in its absence (when only the labeled hormone is bound), respectively.
